# Identification and Nematicidal Characterization of an Extracellular Chitinase BLChi79 from *Brevibacillus laterosporus* Strain XJ-24-3

**DOI:** 10.3390/vetsci13070656

**Published:** 2026-07-07

**Authors:** Shuang Chen, Yikuan Qian, Lixiang Wei, Ming Wu, Yu Yang, Xuepeng Cai, Jie Li, Qingling Meng, Jun Qiao

**Affiliations:** 1 College of Animal Science and Technology, Shihezi University, Shihezi 832003, China; 18181160768@163.com (S.C.); k18332157692@163.com (Y.Q.);; 2 State Key Lab of Veterinary Etiological Biology, Lanzhou Veterinary Research Institute, Chinese Academy of Agricultural Sciences, Lanzhou 730046, China; 3 School of Basic Medicine, Xinjiang Second Medical University, Karamay 834000, China

**Keywords:** *Brevibacillus laterosporus*, chitinase BLChi79, enzymatic characterization, nematode eggs, nematicidal activity

## Abstract

This study aimed to identify and characterize a high-efficiency chitinase gene from *Brevibacillus laterosporus* (*B. laterosporus*), characterize its enzymatic traits, and assess its degrading activity against *Caenorhabditis elegans* (*C. elegans*) and *Parascaris equorum* (*P. equorum*) eggs. A nematicidal *B. laterosporus* isolate was subjected to whole-genome sequencing for chitinase gene screening, cloning, and molecular identification. The target gene was heterologously expressed in *Escherichia coli* BL21 (DE3), and biochemical properties including temperature, pH, metal ions, and substrate conditions were investigated, its kinetic parameters were determined, and its biological effects on *C. elegans* and *P. equorum* eggs were analyzed. The protein BLChi79 (79.3 kDa) belongs to GH18 chitinases with a typical carbohydrate-binding module. Its optimum activity occurred at 60 °C and pH 6.0, with colloidal chitin as the optimal substrate. Mg^2+^, Fe^2+^, and Mn^2+^ boosted its activity, while K^+^, Cu^2+^, Zn^2+^, and Ca^2+^ suppressed catalysis. Its K_m_ was 6.14 mg·mL^−1^, V_max_ 7.78 μmol·min^−1^·mg^−1^, and kcat 10.16 min^−1^. The purified recombinant enzyme could degradethe *C. elegans* chitin layer and the *P. equorum* egg vitelline envelope. In summary, the BLChi79 derived from *B. laterosporus* targets chitin-enriched eggshells, acting as a green biocatalyst for controlling livestock gastrointestinal nematodes.

## Simple Summary

Nematode parasites seriously harm livestock breeding, and traditional chemical nematicides easily cause environmental pollution and drug resistance, so eco-friendly biological control agents are urgently needed. This work screened a chitinase gene *BLChi79* from the genome of the nematode-killing strain *Brevibacillus laterosporus* XJ-24-3. We successfully produced the purified BLChi79 protein and tested its enzymatic features and egg-degrading function. BLChi79 is a GH18 family chitinase that works best at 60 °C and neutral weak acid conditions, and its catalytic activity can be improved by magnesium, iron and manganese ions. Functional tests proved this enzyme can break down the chitin-containing outer shells of two harmful nematode eggs: model worm *Caenorhabditis elegans* and livestock parasite *Parascaris equorum*. Overall, BLChi79 can specifically destroy nematode eggshells, and it has great potential to be developed as a safe, green biocatalyst to control gastrointestinal nematode infections in farm animals.

## 1. Introduction

Gastrointestinal nematode infections represent a globally prevalent parasitic disease affecting herbivorous livestock, resulting in impaired growth, chronic diarrhea, weight loss, and increased mortality, thereby imposing substantial economic burdens on the livestock industry and threatening food security [1,2,3]. Current control strategies rely predominantly on synthetic anthelmintics—including benzimidazoles, imidazothiazoles, and macrocyclic lactones—which are increasingly compromised by widespread anthelmintic resistance in target nematodes, residual contamination in animal products and the environment, and associated risks to human health and ecological stability [4,5]. Consequently, there is an urgent need to develop effective, biodegradable, and non-toxic alternatives grounded in mechanistic biological understanding.

*Brevibacillus laterosporus (B. laterosporus*) is a Gram-positive, spore-forming bacterium recognized for its capacity to synthesize diverse extracellular bioactive metabolites, including chitinases, antimicrobial peptides, and insecticidal crystal proteins [6,7,8]. Accumulating evidence underscores its nematicidal potential; for instance, targeted deletion of blg4—encoding an extracellular alkaline protease in *B. laterosporus* strain G4—resulted in an approximately 50% reduction in larvicidal activity against and markedly attenuated virulence in infection models, confirming BLG4 as a key nematicidal effector [9,10]. Moreover, chitinase-mediated nematicidal activity has been independently documented. De Andrade et al. reported that *B. laterosporus* 1864—a high-chitinase-producing isolate—exhibited significant lethality against second-instar larvae of *Culex pipiens pallens* [8]. Similarly, Miao et al. demonstrated that chitinase from *B. laterosporus* GWM1.8072 effectively suppressed hatching of southern root-knot nematode (*Meloidogyne incognita*) eggs [11].

Although the nematicidal potential of *B. laterosporus* has been established, all reported studies to date have focused on insect or plant-parasitic nematodes, leaving the activity of its chitinase against gastrointestinal nematodes of livestock—particularly the eggs of large animal ascarids—largely unexplored. In this work, we employed a hybrid sequencing strategy combining second- and third-generation platforms to obtain the complete genome of *B. laterosporus* strain XJ-24-3, which was isolated in Xinjiang. From the genomic data, we screened and cloned a putatively high-activity chitinase, designated BLChi79, performed molecular characterization, and, after prokaryotic expression and purification, determined its enzymatic properties. We further evaluated its biological effects on *Caenorhabditis elegans* (*C. elegans*) and *Parascaris equorum* (*P*. *equorum*) eggs. Unlike earlier investigations that relied on crude activity assays and remained at a relatively superficial level, our study proceeds systematically from gene identification and enzymology to biocontrol functionality, thus filling a clear knowledge gap regarding the specific chitinase of *B. laterosporus* against livestock gastrointestinal nematodes. The findings not only provide a theoretical foundation for dissecting the molecular mechanism of nematode antagonism by this chitinase but also offer a basis for developing efficient biological nematicidal enzyme preparations in the future.

## 2. Materials and Methods

### 2.1. Strains and Reagents

*B. laterosporus* XJ-24-3, the pET32a expression vector, *Caenorhabditis elegans* N2 (wild-type), and *Parascaris equorum* eggs were maintained at the Key Laboratory of Preventive Veterinary Medicine, Shihezi University. *Escherichia coli* DH5α and BL21(DE3) competent cells were obtained from Shanghai Sangon Biotech Co., Ltd. (Shanghai, China). The pMD19-T vector, T4 DNA ligase, and restriction endonucleases EcoRI and HindIII were purchased from Dalian Baobio Co., Ltd. (Dalian, China). PCR Master Mix, DNA molecular weight marker, and protein molecular weight marker were acquired from Beijing Quantype Gold Biotechnology Co., Ltd. (Beijing, China).

### 2.2. Preparation of Colloidal Chitin

Colloidal chitin was prepared according to the method described by Pan M et al. (2019) [12], with minor modifications. Briefly, 2 g of chitin powder was suspended in 40 mL of ice-cold concentrated hydrochloric acid (37%, *v*/*v*) and stirred magnetically at 4 °C until complete dissolution yielded a homogeneous, clear, pale-yellow solution. The mixture was sealed and stored at 4 °C for 24 h. Subsequently, 300 mL of pre-chilled 50% (*v*/*v*) ethanol was slowly added dropwise under continuous stirring on ice, resulting in the formation of a milky-white colloidal suspension. The suspension was centrifuged at 9000× *g* for 20 min at 4 °C; the supernatant was discarded, and the pellet was washed repeatedly with ultrapure water until neutral pH (pH 6.8–7.2) was achieved, as confirmed by pH indicator paper. The final colloidal chitin suspension was stored at 4 °C and used within one week.

### 2.3. Genomic DNA Extraction and Whole-Genome Sequencing

Genomic DNA was extracted from *B. laterosporus* XJ-24-3 using the Bacterial Genomic DNA Extraction Kit (Tiangen Biotech, Beijing, China), following the manufacturer’s instructions. High-quality genomic DNA (A_260_/A_280_ ≈ 1.8–2.0; A_260_/A_230_ > 2.0) was subjected to whole-genome sequencing on an Illumina NovaSeq 6000 platform (paired-end, 150 bp reads) by Novogene Co., Ltd. (Beijing, China). Raw reads were trimmed and filtered using FASTP v0. 23. 2 to remove low-quality bases (Q < 20), adapter sequences, and reads shorter than 50 bp [13]. High-quality clean reads were assembled de novo using SPAdes v3.15.5 with default parameters. Genome scaffolds were annotated using the Integrated Microbial Genomes and obiomes (IMG/M) platform [14]. Structural features including genomic islands, prophages, and repetitive elements were analyzed using the IPGA web server [15]. Functional annotation was performed using BLASTP 2.17.0+against the Gene Ontology (GO), Kyoto Encyclopedia of Genes and Genomes (KEGG), Carbohydrate-Active Enzymes (CAZy), Clusters of Orthologous Groups (COG), and Transporter Classification Database (TCDB) resources.

### 2.4. Cloning, Molecular Characterization, and Phylogenetic Analysis of the Chitinase Gene BLChi79

Based on the draft genome sequence, gene-specific primers BLChi79-F (5′-CCGGAATTCATGAAAAGATTTTTCTCATGGC-3′) and BLChi79-R (5′-CCCAAGCTTCTACTGACGTTCTTTAGTAAGT-3′) were designed to amplify the full-length coding sequence of *BLChi79*, incorporating EcoRI and HindIII restriction sites (underlined), respectively. PCR amplification was carried out using high-fidelity DNA polymerase (Phanta Max Super-Fidelity DNA Polymerase, Vazyme). Amplified products were resolved by 1% (*w*/*v*) agarose gel electrophoresis, excised, and purified using a Gel Extraction Kit (Omega Bio-tek). Purified fragments were ligated into the pMD19-T vector (Takara, Japan) at 16 °C overnight and transformed into chemically competent *E. coli* DH5α cells. Positive clones were screened by colony PCR and confirmed by double restriction digestion (EcoRI/HindIII) and Sanger sequencing. Deduced amino acid sequences were analyzed for molecular weight, isoelectric point (pI), and signal peptide prediction using ProtParam (ExPASy, SIB Swiss Institute of Bioinformatics, Lausanne, Switzerland) and SignalP 6.0. Multiple sequence alignment of chitinase homologs was conducted using Clustal Omega (EMBL-EBI); phylogenetic reconstruction was performed via the maximum-likelihood (ML) method in IQ-TREE v2.2.0 with 1000 ultrafast bootstrap replicates. Homology modeling of the catalytic domain was generated using SWISS-MODEL (ExPASy, SIB Swiss Institute of Bioinformatics, Lausanne, Switzerland) (automated mode, template PDB ID: 1E6N), and structural visualization and analysis were performed using PyMOL 2.5.2 (Schrödinger, LLC, Cambridge, MA, USA).

### 2.5. Construction of the Prokaryotic Expression Vector

The *BLChi79* coding sequence was amplified using primers BLChi79-F and BLChi79-R. Both the PCR product and pET32a vector were digested with EcoRI and HindIII, purified, and ligated using T4 DNA ligase at 16 °C overnight. Ligation mixtures were transformed into *E. coli* DH5α competent cells and plated on LB agar supplemented with 100 µg·mL^−1^ ampicillin. Single colonies were cultured, plasmid DNA isolated, and constructs verified by colony PCR and diagnostic double digestion. Sequence integrity of the insert was confirmed by bidirectional Sanger sequencing.

### 2.6. Expression, Purification, and Identification of Recombinant BLChi79 Protein

The verified recombinant plasmid pET32a-BLChi79 was transformed into *E. coli* BL21(DE3) cells. Protein expression was induced with 1 mM isopropyl β-D-1-thiogalactopyranoside (IPTG) at 16 °C for 16 h (to optimize soluble protein expression). Cells were harvested by centrifugation (6000× *g*, 10 min, 4 °C), resuspended in lysis buffer (50 mM Tris-HCl pH 8.0, 300 mM NaCl, 10 mM imidazole), and lysed by sonication. Soluble fractions were applied to Ni^2+^-NTA affinity resin (Qiagen), washed with wash buffer (50 mM Tris-HCl pH 8.0, 300 mM NaCl, 20 mM imidazole), and eluted with elution buffer (50 mM Tris-HCl pH 8.0, 300 mM NaCl, 250 mM imidazole). Eluates were dialyzed against storage buffer (20 mM Tris-HCl pH 7.5, 150 mM NaCl, 10% glycerol) at 4 °C overnight. Protein concentration was determined by Bradford assay. Purity and molecular weight were assessed by SDS-PAGE (12% gel), and identity was confirmed by Western blotting using anti-His tag monoclonal antibody.

### 2.7. Enzyme Activity Assay

Chitinase activity was quantified using the 3,5-dinitrosalicylic acid (DNS) method [16], with N-acetyl-D-glucosamine (NAG) as the standard. A calibration curve (OD_540_ versus NAG concentration, 0–1.0 mM) yielded the linear regression equation y = 2.578x − 0.1300 (R^2^ = 0.9903; Figure S1). One unit (U) of chitinase activity was defined as the amount of enzyme required to liberate 1 µmol of NAG per minute under standard assay conditions (pH 6.0, 40 °C, 30 min reaction). Reaction mixtures contained 50 µL of appropriately diluted enzyme, 150 µL of 1% (*w*/*v*) colloidal chitin in 50 mM sodium phosphate buffer (pH 6.0), and were incubated at 40 °C. After termination by boiling for 5 min, 200 µL of DNS reagent was added, followed by heating at 100 °C for 5 min. Absorbance at 540 nm was measured after cooling to room temperature. Each assay included triplicate technical replicates and a blank control (heat-inactivated enzyme). Specific activity was expressed as U·mg^−1^ protein.

### 2.8. Biochemical Characterization of Recombinant BLChi79

The optimal temperature was determined by measuring activity across a range of 25–70 °C (in 5 °C increments) under standard conditions; relative activity was calculated with respect to the maximum value (set to 100%). Thermostability was assessed by pre-incubating enzyme solutions (1 mg·mL^−1^ in 50 mM sodium phosphate, pH 6.0) at selected temperatures (30–70 °C) for 60 min, followed by immediate cooling on ice and residual activity measurement at 40 °C. Optimal pH was evaluated using 50 mM buffers: glycine-HCl (pH 3.0–5.0), sodium phosphate (pH 5.0–7.0), Tris-HCl (pH 7.0–9.0), and glycine-NaOH (pH 9.0–10.0). pH stability was determined by incubating the enzyme (1 mg·mL^−1^) in each buffer for 60 min at 4 °C, followed by a residual activity assay at optimal pH and temperature. The effects of metal ions (Na^+^, Mg^2+^, K^+^, Fe^2+^, Zn^2+^, Cu^2+^, Ca^2+^, Mn^2+^, Ba^2+^) were tested at final concentrations of 1 mM and 5 mM; activity in the absence of added ions served as the control (100%). Substrate specificity was examined using 1% (*w*/*v*) colloidal chitin, crystalline chitin, chitosan (deacetylation degree > 85%), carboxymethyl cellulose sodium salt (CMC-Na), dextrin, and soluble starch. Kinetic parameters were determined using colloidal chitin at concentrations ranging from 5 to 40 mg·mL^−1^ in 50 mM sodium phosphate buffer (pH 6.0) at 40 °C. Initial velocities were fitted to the Michaelis–Menten equation using GraphPad Prism 9.5.0; K_m_, V_max_, and kcat (=V_max_/[E]) were derived, where [E] denotes the molar concentration of active enzyme.

### 2.9. Nematicidal Activity Against C. elegans

Synchronized *C. elegans* N2 larvae (L4 stage) and young adults were harvested from NGM plates using M9 buffer, pelleted (2000× *g*, 2 min), and resuspended to yield approximately 50 nematodes per 5 µL. In a sterile 96-well plate, 100 µL of purified recombinant BLChi79 (1 mg·mL^−1^), 5 µL nematode suspension, and 1.2 µL chloramphenicol (50 mg·mL^−1^, final concentration 0.5 µg·mL^−1^ to suppress bacterial growth) were mixed per well. Wells containing heat-inactivated recombinant BLChi79 (boiled for 10 min at 100 °C) served as negative controls. Plates were sealed with parafilm and incubated at 20 °C. Morphological integrity and cuticle degradation of nematodes were monitored at 2, 6, 12, 24, and 48 h post-treatment using an inverted microscope (Olympus IX73).

### 2.10. Ovicidal Activity and Scanning Electron Microscopy (SEM) Analysis Against P. equorum Eggs

*P. equorum* eggs were isolated from equine feces via sugar flotation, washed three times with cold PBS (3000× *g*, 2 min, 4 °C), and resuspended in PBS to approximately 100 eggs per 5 µL. In a sterile 96-well plate, 100 µL recombinant BLChi79 (1 mg·mL^−1^), 5 µL egg suspension, and 1.2 µL chloramphenicol (50 mg·mL^−1^) were added per well. Heat-inactivated enzyme served as the control. Plates were incubated at 37 °C, and egg morphology was observed at 6, 12, 24, 48, and 72 h. For SEM analysis, eggs from treated and control groups were collected by centrifugation (3000× *g*, 2 min), fixed in 2.5% glutaraldehyde in 0.1 M phosphate buffer (pH 7.4) for 12 h at 4 °C, post-fixed in 1% osmium tetroxide, dehydrated through graded ethanol series (30–100%), subjected to critical-point drying (Leica EM CPD 300, Tokyo, Japan), and sputter-coated with gold (Leica EM ACE 600, Tokyo, Japan). Surface ultrastructure was visualized using a Hitachi SU8220 SEM (Tokyo, Japan) operated at 2 kV accelerating voltage and ×800 magnification.

### 2.11. Statistical Analysis

All experiments were performed in triplicate (biological replicates). Data are presented as mean ± standard deviation (SD). Statistical significance was assessed by one-way ANOVA with Tukey’s post hoc test (α = 0.05) using GraphPad Prism 9.5.0. Figures were generated using the same software.

## 3. Results

### 3.1. Whole-Genome Sequencing of B. laterosporus BL-XJ-24-3

**Figure 1:**
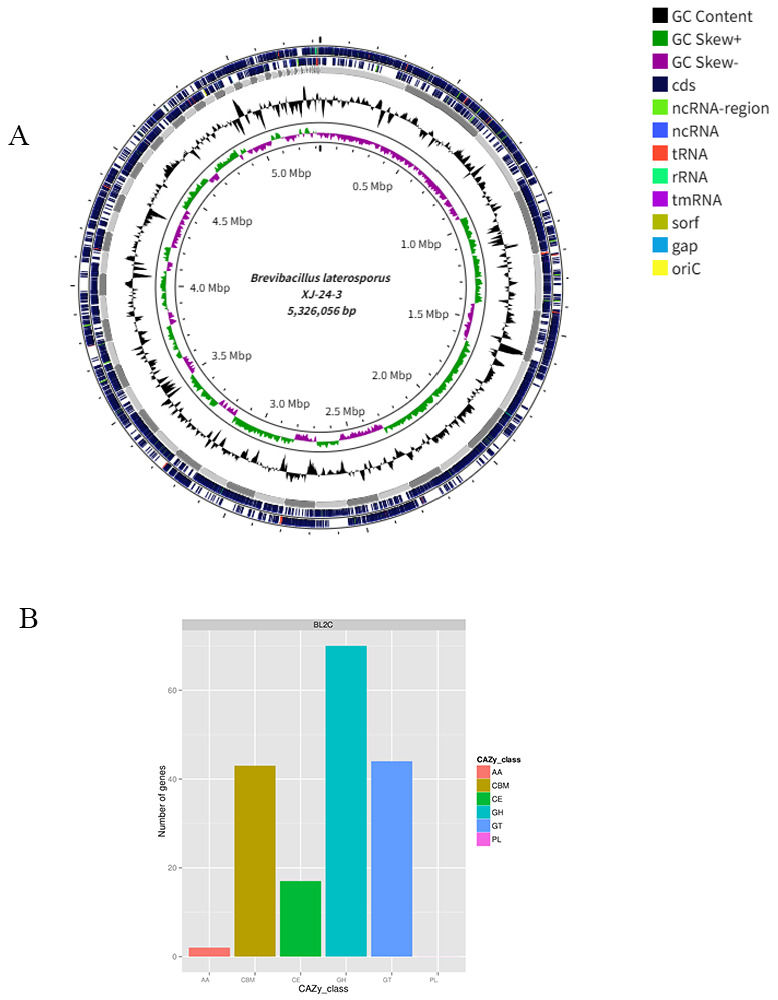
Genome sequencing and map of the *Brevibacillus laterosporus.* (**A**) Genome map of the *Brevibacillus laterosporus XJ-24-3*. Note: From inside to outside: the first circle represents the scale; the second circle represents GC skew; the third circle represents the GC content; the fourth and seventh circles represent the COG to which each CDS belongs; and the fifth and sixth circles represent the positions of CDS, tRNA, and rRNA on the genome. (**B**) Carbohydrate-active enzyme (CAZy) function prediction.

The complete genome of *B. laterosporus XJ-24-3* comprises 5,326,056 base pairs (bp), with an N_50_ contig length of 122,366 bp and a GC content of 40.3%, Gene prediction identified 5039 protein-coding sequences, collectively spanning 4,522,398 bp, yielding an average coding sequence length of 897 bp. Comparative genomic analysis revealed 13 genomic islands and 29 prophage regions. Non-coding RNA annotation detected 106 transfer RNAs (tRNAs) and eight small RNAs (sRNAs), as visualized in the circular genome map (Figure 1A). Additionally, the genome harbors 261 dispersed repetitive elements.

### 3.2. Functional Annotation and Chitinase Gene Mining

Functional annotation of the *B. laterosporus XJ-24-3* genome was performed using multiple public databases. The NCBI non-redundant (NR) database provided the highest annotation coverage, assigning putative functions to 4804 genes. Within the CAZy database classification, chitinases are categorized into two glycoside hydrolase (GH) families: GH18 and GH19 [17]. A total of 70 GH-family genes were identified in the *B. laterosporus XJ-24-3* genome (Figure 1B). Subsequent BLASTP analysis against the NCBI nr database identified two genes encoding canonical chitinases—designated *BLChi79* and *BLChi4036* based on domain architecture, conserved motifs, and sequence homology.

### 3.3. Cloning and Bioinformatic Analysis of BLChi79 Gene

**Figure 2:**
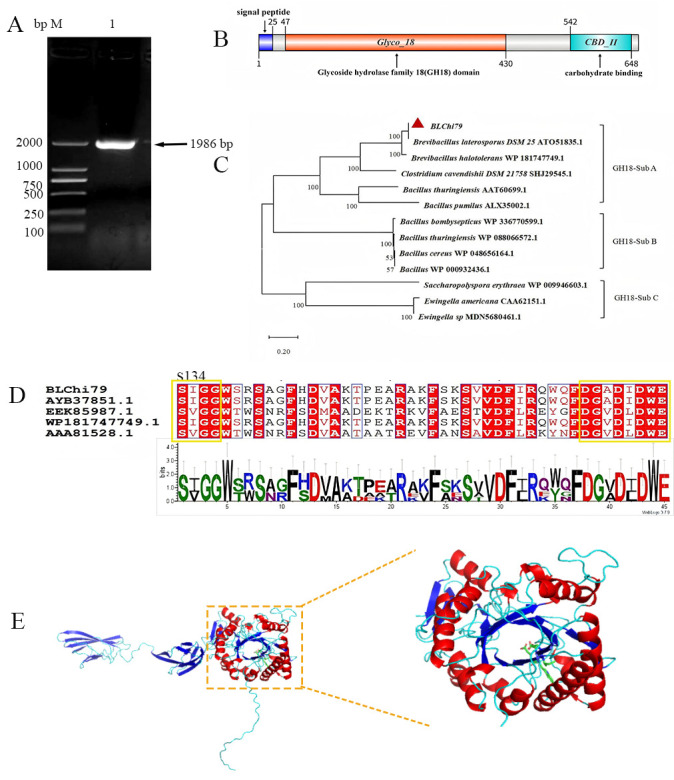
Molecular characteristics of chitinase BLChi79 from *Brevibacillus laterosporus*. (**A**) BLChi79 gene PCR amplification electrophoresis product. M: DNA marker DL-2000 (2000, 1000, 750, 250, 100 bp); 1: PCR amplification product of *BLChi79* gene. (**B**) Structural domain pattern diagram of chitinase BLChi79. (**C**) Genetic evolution analysis of chitinase BLChi79. (**D**) Multisequence comparison of the partial patalytic domain of chitinase BLChi79.Note:The yellow box represents the conservative motif (**E**) Protein structure diagram of BLChi79; red indicated α-helix, blue indicated β-fold.

A 1986-bp amplicon corresponding to the full-length *BLChi79* coding sequence was amplified from genomic DNA of *B. laterosporus XJ-24-3* using gene-specific primers (Figure 2A). The mature open reading frame (ORF) spans 1968 bp and encodes a 661-amino-acid polypeptide featuring an N-terminal signal peptide. Domain architecture analysis (Figure 2B) confirmed the presence of a catalytic Glyco_18 domain (residues 45–430) and a C-terminal carbohydrate-binding module (CBM type II; residues 542–648). Phylogenetic reconstruction placed BLChi79 unambiguously within subfamily A of the GH18 family (Figure 2C). Conserved motif analysis further validated hallmark features of GH18 chitinases: the chitin-binding motif SxGG and the catalytic triad DxxDxxDxE (Figure 2D). Three-dimensional structural modeling revealed that the catalytic domain adopts the canonical (β/α)_8_ TIM-barrel fold, with characteristic α+β insertions consistent with subfamily A architecture (Figure 2E). Nucleotide sequence identity comparisons indicated 99.85%, 90.14%, and 89.83% identity to orthologous genes from *B. laterosporus* strains DSM 25, BL-ZJ, and LMG 15441, respectively.

### 3.4. SDS-PAGE and Western Blot Validation of Recombinant BLChi79

**Figure 3:**
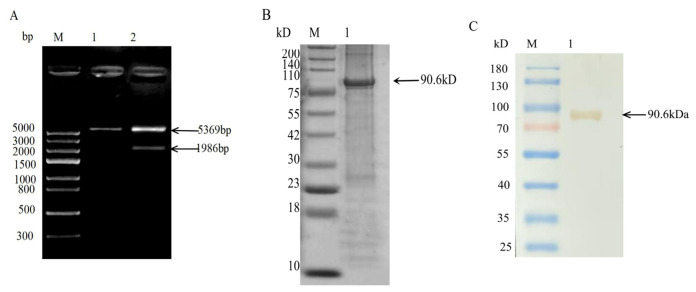
Expression of the chitinase gene *BLChi79* of *Brevibacillus laterosporus*. (**A**) Identification of recombinant plasmid pET32a-BLChi79 by restriction endonuclease double digestion. M: DNA marker DL-8000 (8000, 5000, 3000, 1500, 1000, 500, 300 bp); 1: blank control; 2: recombinant plasmid pET32a-BLChi79 conducted by double enzyme digestion. (**B**) Expression and purification of BLChi79 by SDS-PAGE analysis; M: protein maker (10~200 kD); 1: purified chitinase BLChi79. (**C**) identification of purified BLChi79 using Western blot. M: protein maker (10~180 kDa); 1: purified chitinase BLChi79.

Successful construction of the pET32a-BLChi79 expression plasmid was confirmed by double restriction digestion with EcoRI and HindIII, yielding fragments of expected size (Figure 3A). SDS-PAGE analysis demonstrated robust expression of soluble recombinant protein at approximately 90.6 kDa (including the approximately 17.3-kDa pET32a His-tag fusion), consistent with the theoretical molecular weight of the full-length fusion protein (Figure 3B). Western blotting using anti-His antibodies confirmed specific detection of the recombinant protein at the same molecular weight (Figure 3C).

### 3.5. Analysis of Enzymatic Properties of Recombinant Chitinase BLChi79

**Figure 4:**
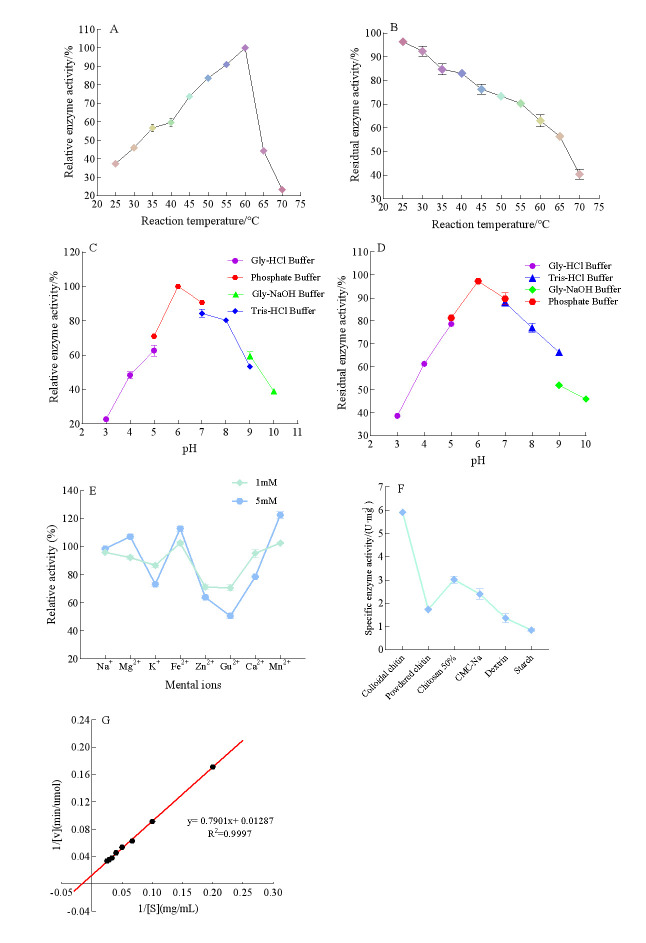
Determination of enzymatic properties of chitinase BLChi79. (**A**) The optimal reaction temperature for chitinase BLChi79. (**B**) Temperature stability of chitinase BLChi79. (**C**) The optimal pH for chitinase BLChi79. (**D**) pH stability of chitinase BLChi79. (**E**) The effect of different metal ions on the activity of chitinase BLChi79. (**F**) Substrate specificity of chitinase BLChi79. (**G**) Kinetic parameters of chitinase BLChi79.

BLChi79 exhibited maximal activity at 60 °C, retaining ≥83.7% relative activity between 50–60 °C and declining to 44.3% at 65 °C. Thermal stability assays revealed >70% residual activity following 1 h incubation at 25–55 °C; activity remained at 61.7% after incubation at 60 °C (Figure 4A,B). The enzyme displayed optimal activity at pH 6.0 and maintained >62.3% relative activity across pH 5.0–8.0. Following 1 h incubation across this pH range, residual activity exceeded 77%, indicating broad pH stability (Figure 4C,D). Metal ion profiling demonstrated that 1 mM Fe^2+^ and Mn^2+^ slightly enhanced activity (approximately 102% relative activity), whereas Mg^2+^, Fe^2+^, and Mn^2+^ at 5 mM exerted stronger stimulatory effects; Cu^2+^ strongly inhibited activity (Figure 4E). Substrate specificity analysis demonstrated highest hydrolytic efficiency toward colloidal chitin (5.90 U·mg^−1^; Figure 4F). Kinetic parameters derived from Michaelis–Menten equation analysis yielded a Michaelis constant (K_m_) of 6.14 mg·mL^−1^ and a maximum velocity (V_max_) of 7.78 μmol·min^−1^·mg^−1^, corresponding to a catalytic turnover number (kcat) of 10.16 min^−1^ (Figure 4G).

### 3.6. Nematicidal Activity of Recombinant BLChi79 Against C. elegans

**Figure 5:**
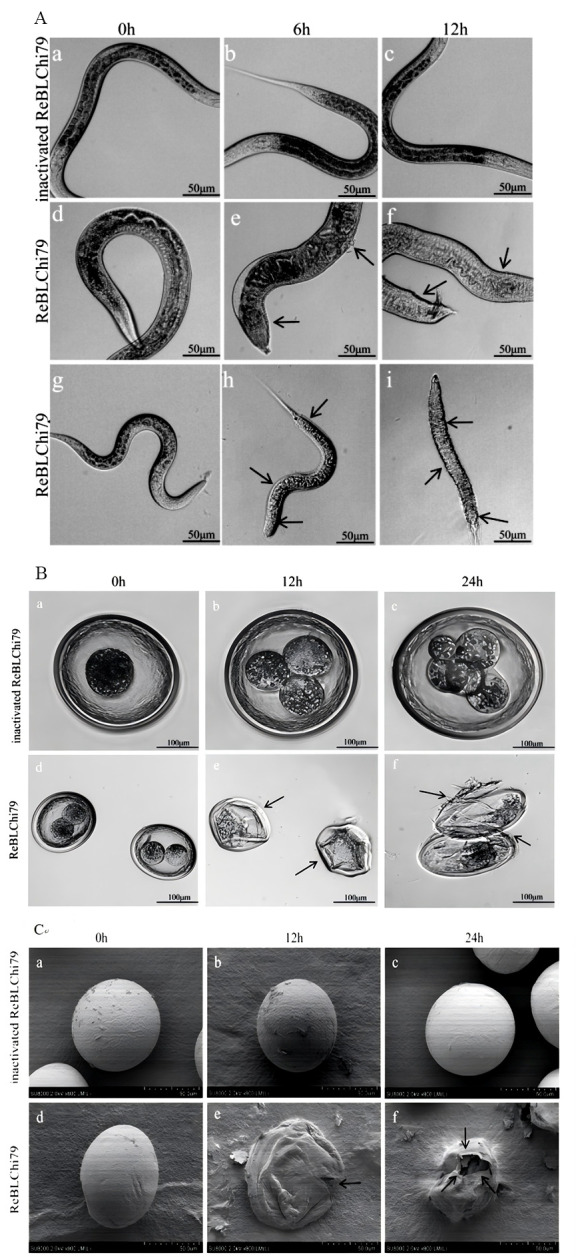
Determination of nematocidal activity of recombinant chitinase ReBLChi79 from *Brevibacillus laterosporus*. (**A**) The degradation activity of ReBLChi79 on *Caenorhabditis elegans*. (**a**–**c**): *C. elegans* treated with inactivated ReBLChi79 for 0 h, 6 h, and 12 h, respectively; (**d**–**f**): Adult *C. elegans* treated with ReBLChi79 for 0 h, 6 h, and 12 h, respectively; (**g**–**i**): Stage-III larvae of *C. elegans* treated with ReBLChi79 for 0 h, 6 h, and 12 h, respectively. Note: Arrow-degraded part of the nematode body wall (scale bar–50 µm). (**B**) The degradation of *Parascaris equorum* eggs by the ReBLChi79. (**a**–**c**): *Parascaris equorum* eggs treated with inactivated ReBLChi79 for 0 h, 12 h, and 24 h, respectively; (**d**–**f**): *Parascaris equorum* eggs treated with ReBLChi79 for 0 h, 12 h, and 24 h, respectively. Note: Arrow-degraded part of the *Parascaris equorum* eggs (scale bar–100 µm). (**C**) The *Parascaris equorum* eggs treated by ReBLChi79 using a scanning electron microscope (Hitachi High-Tech Corporation, Tokyo, Japan). (**a**–**c**): *Parascaris equorum* eggs treated with inactivated ReBLChi79 for 0 h, 12 h, and 24 h, respectively; (**d**–**f**): *Parascaris equorum* eggs treated with ReBLChi79 for 0 h, 12 h, and 24 h, respectively. Note: Arrow-degraded part of the *Parascaris equorum* eggs (scale bar–50 µm).

Exposure to recombinant BLChi79 induced progressive cuticle degradation in *C. elegans*. At 6 h post-treatment, morphological alterations—including cuticle shrinkage and localized disintegration—were observed in 70% of adult and 89% of larval nematodes. By 12 h, severe structural collapse affected 95% of adults and 100% of larvae, characterized by extensive cuticle rupture, loss of body integrity, and dissolution of internal contents (Figure 5(Aa–c)). In contrast, untreated controls retained smooth, intact cuticles. Time-resolved analysis revealed that initial degradation in adults commenced predominantly in the ventral abdominal region (Figure 5(Ad–f)); by 12 h, widespread fragmentation and internal content leakage were evident. Larvae exhibited early degradation at the cephalic and caudal extremities (6 h), with progressive cuticle shedding, severe body contraction, and extrusion of internal tissues by 12 h (Figure 5(Ag–i)).

### 3.7. Ovicidal Activity of Recombinant BLChi79 Against P. equorum Eggs

Treatment with recombinant BLChi79 arrested embryonic development in *P. equorum* eggs. After 12 h, embryos appeared disorganized within deformed eggshells (Figure 5(Be)). By 24 h, eggshells were fractured and degraded, with complete loss of internal contents and gross morphological distortion (Figure 5(Bf)). The control eggs remained structurally intact, with normally developing embryos (Figure 5(Ba–c)). Scanning electron microscopy confirmed these observations: the control eggshells retained smooth, dense, and continuous surfaces over 24 h (Figure 5(Ca–c)), whereas the treated samples exhibited pronounced surface collapse, irregular folding, and depression after 12 h (Figure 5(Ce)). At 24 h, extensive shell erosion, multifocal cracking, and pore formation exposed underlying structures, accompanied by leakage and degradation of intrauterine material (Figure 5(Cf)).

## 4. Discussion

Gastrointestinal nematode control in livestock currently relies predominantly on chemical anthelmintic agents. However, limitations including anthelmintic resistance, food safety concerns, and environmental pollution have become increasingly prominent. Therefore, there is an urgent need to identify safe and effective biological control strategies for nematode infections in herbivorous animals. In recent years, *B. laterosporus* has been found to produce diverse extracellular hydrolytic enzymes (proteases, chitinases, collagenases, lipases), which can degrade nematode cuticles and eggshells [6,7], suggesting its potential as a candidate organism for development of biocontrol agents.

Chitin is a biopolymer composed of N-acetyl-D-glucosamine units connected by β-1,4-glycosidic bonds. It constitutes an essential structural component of the cuticles of crustaceans, insects, and nematodes, providing supportive and protective functions [18,19,20]. Chitinases are hydrolytic enzymes that catalyze the cleavage of β-1,4-glycosidic bonds in chitin and are widely distributed across diverse organisms, where they participate in multiple physiological processes [21]. Chitinases Chi43 from *Pochonia chlamydosporia* have been demonstrated to degrade the eggs of *Globodera pallida* [22], and chitinases from *Lecanicillium antillanum* have been shown to accelerate the softening of southern root-knot nematode (*Meloidogyne incognita*) eggs, thereby degrading the eggshell and interfering with nematode hatching [23]. Here, we analyzed the molecular characteristics of the chitinase BLChi79 from the *B. laterosporus* XJ-24-3 strain. We confirmed that the chitinase contains a Glyco_18 domain characteristic of the glycoside hydrolase 18 family and a carbohydrate-binding module (CBM type II) in an S+GH18E+CBM arrangement, firmly establishing its classification within the glycoside hydrolase 18 family. Additionally, bioinformatics analysis revealed that BLChi79 contains a signal peptide sequence and is a secreted extracellular chitinase.

Elucidating the enzymatic characteristics of chitinase from *B. laterosporus* is of considerable significance for understanding its biological function and potential applications. Chitinases from different microbial sources exhibit variable temperature optima. For example, chitinases from *Pseudoalteromonas aurantia* display an optimal temperature of 60 °C [24], whereas chitinases from *Chitiniphilus shinanonensis* show an optimal temperature of 50 °C [25]. Wang et al. cloned and expressed the chitinase from *Shewanella khirikhana* JW44, which exhibited an optimal temperature of 40 °C, with residual enzyme activity declining to only 40% after 1 h incubation at 50 °C [26]. In contrast, the recombinant chitinase BLChi79 characterized in this study not only exhibits a relatively high optimal reaction temperature of 60 °C but also demonstrates outstanding thermostability. After incubation for 1 h over a broad temperature range of 25–55 °C, the residual enzyme activity remained above 70%; even when incubated at its optimal temperature of 60 °C for 1 h, it still retained 61.7% of its initial activity. Compared with most bacterial chitinases reported to date, BLChi79 shows markedly superior high-temperature adaptability and thermal stability, enabling it to withstand temperature fluctuations encountered during formulation preparation, storage, and field application. This effectively overcomes the limitations of existing biocontrol chitinases, which generally suffer from poor thermostability and restricted application scenarios, and substantially enhances the practical utility of enzyme preparations derived from *B*. *laterosporus*.

Chitinases undergo varying degrees of ionization at different hydrogen ion (H^+^) concentrations, which affects the S–S bonds, hydrophobic interactions, and hydrogen bonds in their tertiary structure, thereby influencing the conformational stability of chitinases and resulting in differential enzymatic activities [27,28,29]. Chitinases from diverse sources demonstrate considerable variation in pH adaptability, with reported optimal pH ranges spanning from 3.0 to 10.0 [30,31,32]. Wang et al. [33] cloned and expressed the chitinase ChiA derived from *Pseudoalteromonas* sp. DL-6, which exhibited an optimal pH of 9.0 and demonstrated stability under alkaline conditions (pH 8.0–12.0). Sondes Mechri et al. [34] reported that the chitinase from *Nocardiopsis halophila* TN-X8 exhibited maximum enzymatic activity at pH 3.0 and remained relatively stable under acidic conditions. The chitinase BLChi79 identified in this study is a neutral chitinase, with an optimal pH of 6.0, and demonstrates good stability under neutral conditions within the pH range of 6.0–8.0. The gastrointestinal tracts of domestic livestock, as well as aquaculture water bodies and soil environments, are generally neutral to slightly acidic or slightly alkaline in nature. The pH compatibility of BLChi79 is thus highly congruent with these actual application scenarios. Compared with chitinases that exhibit a preference for extreme pH values, BLChi79 possesses superior environmental adaptability, rendering it more suitable for field application in the control of livestock nematodiases.

Metal ions participate in substrate binding and electrostatic stabilization, thereby facilitating enzyme catalysis through stabilization of the enzyme–substrate complex [34]. Studies on *Penicillium oxalicum* revealed that metal ions Zn^2+^, Cu^2+^, and Fe^3+^ inhibited chitinase rChiA-DP activity, whereas Ba^2+^, K^+^, and Li^+^ promoted it [35]. In *Thermomyces lanuginosus*, Cu^2+^ was found to inhibit recombinant chitinase activity [36]. In this study, Mg^2+^, Fe^2+^, and Mn^2+^ promoted the enzymatic activity of BLChi79, whereas K^+^, Cu^2+^, Zn^2+^, and Ca^2+^ inhibited enzymatic activity to varying degrees. Consistent with most chitinases, BLChi79 demonstrated strict substrate specificity, exhibiting significantly higher enzyme activity when using colloidal chitin as substrate compared to other substrates, suggesting that this enzyme plays an important functional role in the degradation of nematode structures. Furthermore, chitinase BLChi79 was demonstrated to degrade the cuticles of both *C. elegans* adults and larvae. The enzyme also exhibited pronounced degradative effects on the eggshells of *P. equorum*, although the degradation rate of the eggshell was slightly slower than that of the cuticle, attributable to differences in structural complexity and composition.

## 5. Conclusions

In this study, we first characterized and analyzed the molecular properties of BLChi79 and determined its enzymatic characteristics through comprehensive biochemical assays. The recombinant chitinase BLChi79 demonstrated potent degradative activity against both the cuticles of *C. elegans* and the eggshells of *P. equorum*, providing an experimental foundation for the development of efficient, environmentally sustainable enzymatic nematicides based on chitinase from *B. laterosporus*. For subsequent phases of this study, it is necessary to evaluate the efficacy of BLChi79 or its enzyme-producing strains in controlling the actual infection pressure exerted by major pathogenic nematodes—such as *Haemonchus contortus* and *Parascaris equorum*—in both natural fecal matter and within definitive hosts herbivorous livestock.

## Data Availability

The original contributions presented in this study are included in the article/Supplementary Materials. Further inquiries can be directed to the corresponding authors.

## Supplementary Materials

The following supporting information can be downloaded at: https://www.mdpi.com/article/10.3390/vetsci13070656/s1, Figure S1: NAG standard curve; Figure S2: Differential expression of chitinase.
