# Supplementary figures and images for: Identification and Nematicidal Characterization of an Extracellular Chitinase BLChi79 from Brevibacillus laterosporus Strain XJ-24-3
Source: Vet Sci. 2026 Jul 7;13(7):656. doi: 10.3390/vetsci13070656 (PMC13431478; doi:10.3390/vetsci13070656)

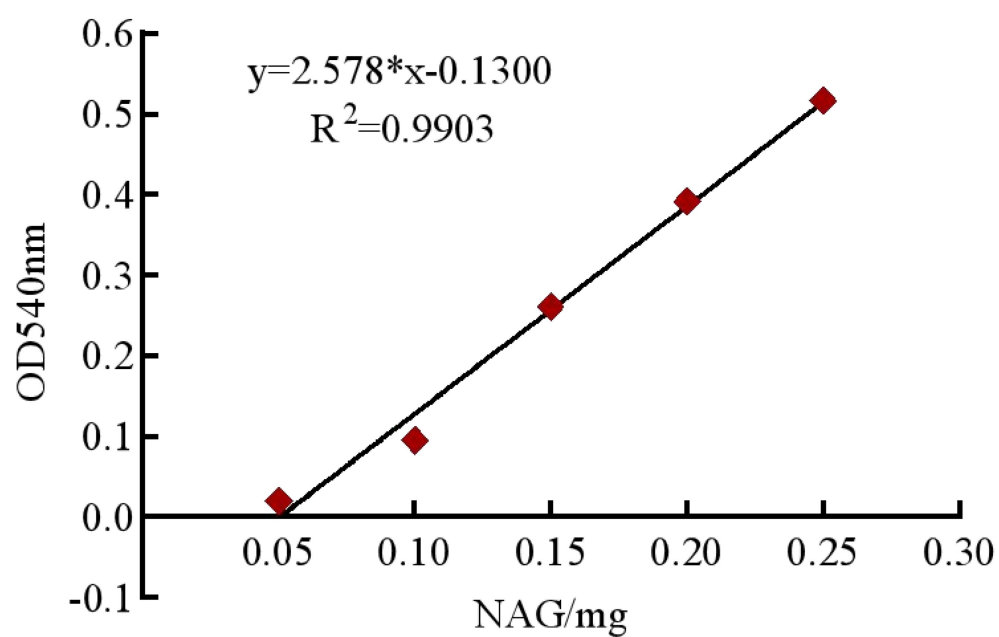

Figure S1. NAG standard curve

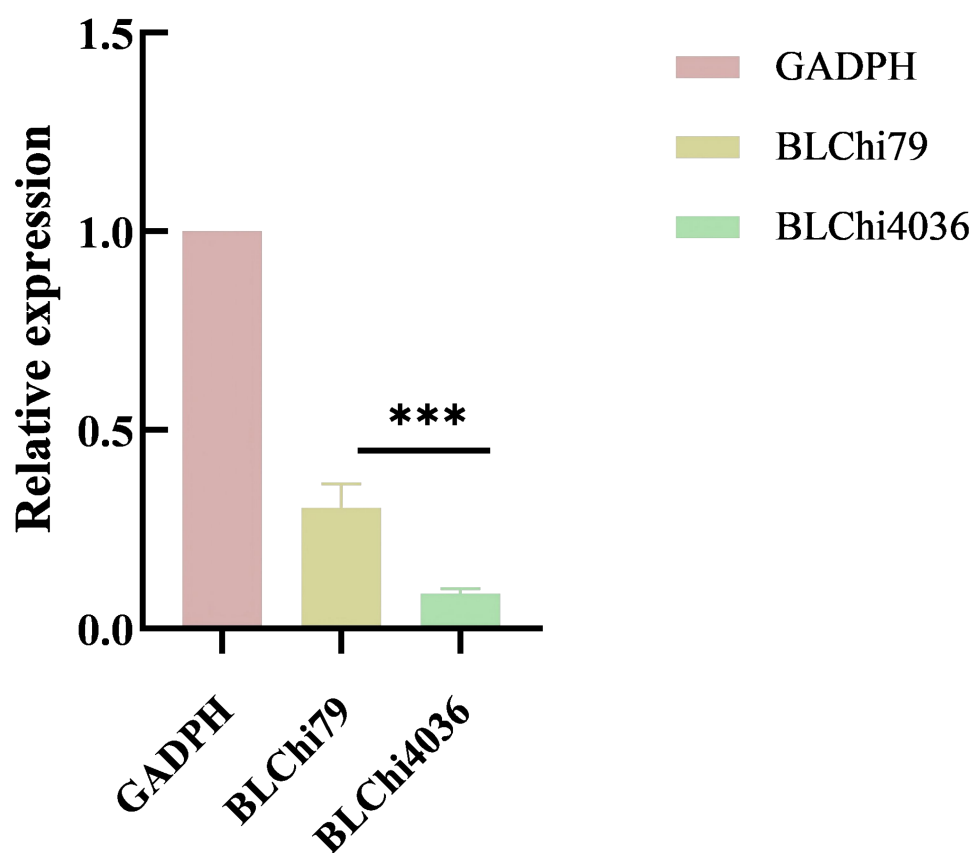

Figure S2. Differential expression of chitinase

Supplement: Supplementary file 1 [file vetsci-13-00656-s001.zip › vetsci-4357981-supplementary-1.pdf]

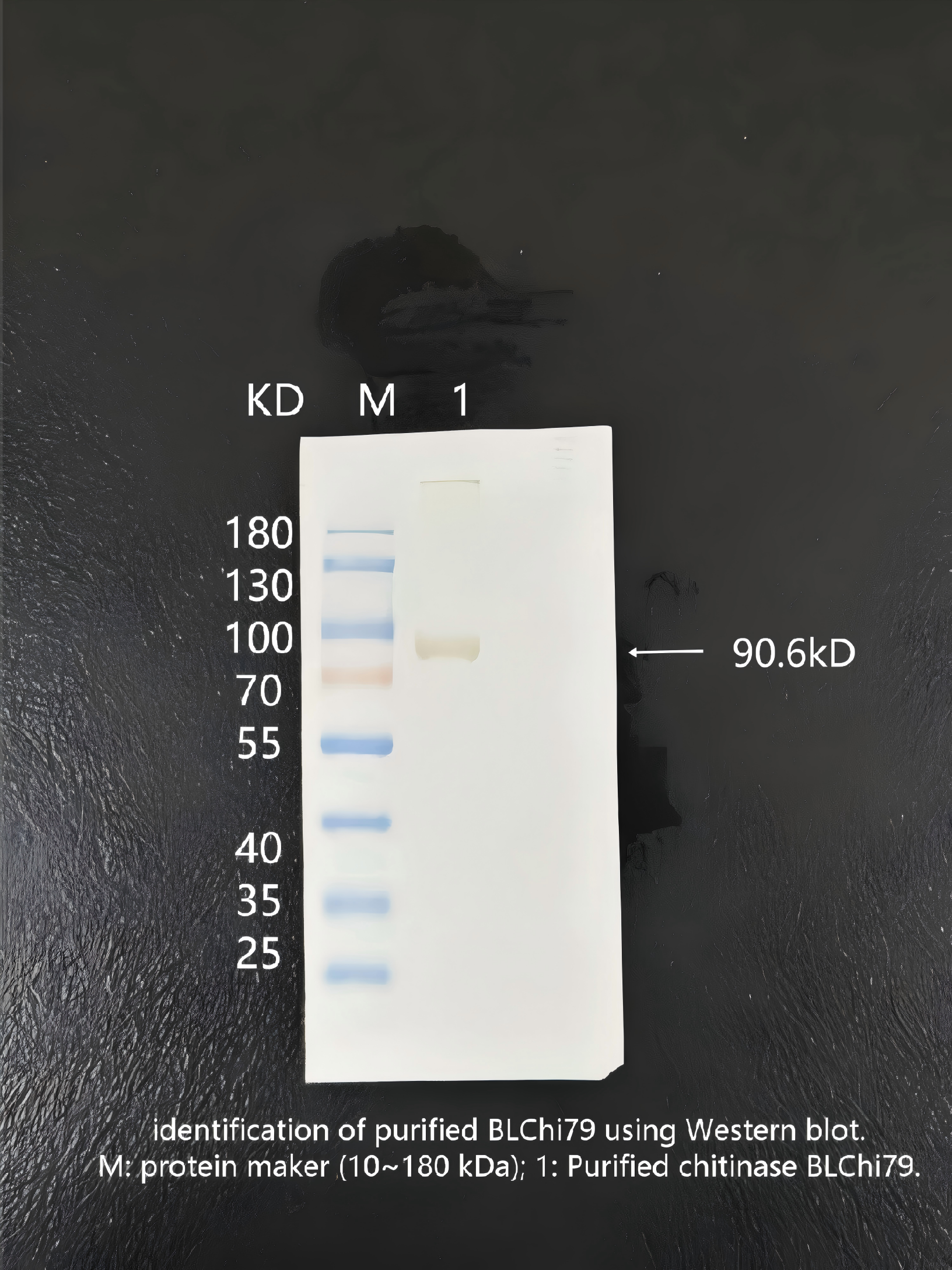

Supplement: Supplementary file 1 [file vetsci-13-00656-s001.zip › wb_original image.png]
